# Occupational support following arthroplasty of the lower limb (OPAL): trial protocol for a UK-wide phase III randomised controlled trial

**DOI:** 10.1136/bmjopen-2024-085962

**Published:** 2024-09-16

**Authors:** Lucy Sheehan, Amie Woodward, Marion Archer, Carol Jordan, Maisie Martland, David A McDonald, Gill Parkinson, Lou Watkins, Joy Adamson, Avril Drummond, Ann Hewison, Ada Keding, Lucksy Kottam, Ira Madan, Catriona McDaid, Mike Reed, Lesley Sinclair, Toby O Smith, Louise Thomson, Qi Wu, Paul Baker

**Affiliations:** 1 Department of Health Sciences, University of York, York, UK; 2 Patient and Public Involvement representative, York, UK; 3 Scottish Government and Social Care Directorates, Edinburgh, UK; 4 University of Nottingham, Nottingham, UK; 5 School of Health Sciences, University of Nottingham, Nottingham, UK; 6 Department of Orthopaedics, South Tees Hospitals NHS Foundation Trust, Middlesbrough, UK; 7 Guys And St Thomas NHS Foundation Trust, London, UK; 8 York Trials Unit, University of York, York, UK; 9 Northumbria Healthcare NHS Foundation Trust, North Shields, UK; 10 Warwick Clinical Trials Unit, University of Warwick, Coventry, UK

**Keywords:** Patients, SURGERY, Knee, Hip, Clinical Trial

## Abstract

**Background:**

In the UK, one in four patients are in work at the time of their hip or knee replacement surgery. These patients receive little support about their return to work (RTW). There is a need for an occupational support intervention that encourages safe and sustained RTW which can be integrated into National Health Service practice. We developed a two-arm intervention trial, based on a feasibility study, to assess whether an occupational support intervention (the OPAL (Occupational support for Patients undergoing Arthroplasty of the Lower limb) intervention) is effective in supporting a reduced time to full, sustained RTW compared with usual care in patients undergoing hip and knee replacement.

**Methods and analysis:**

This is a multicentre, individually randomised controlled superiority trial comparing the OPAL intervention to usual care. 742 working adults listed for elective primary hip or knee replacement, who intend to RTW, will be randomised to the OPAL intervention or usual care. The intervention comprises: (1) multimedia information resources; and (2) support from a designated RTW coordinator. The primary outcome is time until ‘full’ sustained RTW without sick leave for a consecutive 4-week period. Secondary outcomes are: time to any RTW, measures of functional recovery, number of ‘sick days’ between surgery and ‘full’ sustained RTW and the use of workplace modifications to facilitate their return. A health economic evaluation and a mixed methods process evaluation will assess cost-effectiveness and the implementation, fidelity and acceptability of the intervention, respectively. Outcomes will be collected at baseline, 3, 6, 9 and 12-month follow-up time points, as well as a monthly RTW questionnaire.

**Ethics and dissemination:**

Dissemination will focus on supporting the wider adoption and implementation of the intervention (if effective) and will target groups for whom the results will be relevant. This trial was approved by West Midlands—Edgbaston REC 23/WM/0013.

**Trial registration number:**

ISRCTN13694911.

**STRENGTHS AND LIMITATIONS OF THIS STUDY**

- The OPAL trial is testing an evidence-based intervention developed as part of earlier National Institute for Health and Care Research funded research (the OPAL feasibility study health technology assessment 15/28/02, ISRCTN27426982).
- Pragmatic inclusion criteria will allow all patients, in both paid and unpaid work, who intend to return to work (RTW) after surgery, to participate.
- Embedded process evaluation will ensure issues with implementation and adoption are recognised within the trial and used to refine the RTW coordinator role prior to any wider National Health Service adoption at the end of the trial.
- There is a training burden and a requirement for clinical time for the RTW coordinator role which will likely impact on trial delivery given existing clinical pressures on elective services.

## Introduction

### Background and rationale

 In the UK, National Institute for health and Care Excellence (NICE) clinical guidance (NG157) recommends that orthopaedic teams discuss and provide information on return to work (RTW) for patients undergoing primary hip and knee replacement.^1^ Despite this, there is substantial variation in the provision of occupational advice and support.^2^ The current ‘standard of care’ is for patients to receive little or no information or support from their hospital orthopaedic team or General Practitioner (GP) to enable RTW.^3^,^6^ Furthermore, fewer than 35% of patients have access to occupational health support at work.^3^,^6^ There is a need for an occupational support intervention that encourages safe and sustained RTW and can be integrated into UK National Health Service (NHS) practice.

Planned surgery to replace a hip or knee joint is a routine NHS procedure that is becoming more common in people of working age. A quarter of UK patients undergoing primary hip and knee replacement are in work at the time of surgery (~50 000/year).^3 7 8^ This proportion will rise over the next decade with an increasing incidence of hip and knee osteoarthritis^9^ and people working longer.^10^ Returning to work is an important indicator of functional rehabilitation and quality of life. Working has physical and mental health benefits and aids recovery after joint replacement.^11^,^13^ Estimates for the mean time to RTW after hip or knee replacement range from 10 to 14 weeks.^314^,^17^ This equates to approximately 4.2 million days of sickness absence related to recovery after surgery at a societal cost of approximately £400 million/year.^3^

Advice from health professionals about the expected time to RTW can influence absence duration. Patients’ expectations of RTW before surgery are a predictor of work outcomes post-surgery. It is therefore important to provide appropriate advice and support to help patients set realistic expectations about their RTW after surgery. Encouraging and supporting RTW through an occupational intervention initiated prior to surgery could help minimise some of the health and socioeconomic consequences of joint replacement surgery. Determinants of RTW are rarely considered when advising patients about hip and knee replacement surgery and their subsequent RTW.^2 4 5^ In a UK survey, only 19% of health professional respondents routinely offered RTW advice to this patient group and <10% used written information or offered onward referral to occupational health services.^2^ There is therefore significant scope to improve current practice in-line with NICE recommendations (NG157).^1^

In 2016, the health technology assessment (HTA) programme funded a feasibility study conducted by our group (HTA:15/28/02).^3^ This study developed an occupational support intervention for working adults initiated prior to elective hip and knee replacement, to improve the speed of recovery to usual activities including work. The OPAL feasibility study used a mixed-methods research design within an intervention mapping framework to develop an occupational intervention to support RTW after hip and knee replacement. The intervention had a strong theoretical background and was underpinned by biopsychosocial methods that supported behavioural change in the target groups (patients, health professionals and employers). It was manualised as a set of patient and health professional performance objectives that defined its content, format, delivery and timing while maintaining pragmatism in the ability of participating sites to administer the intervention alongside usual care.

The OPAL occupational support intervention consists of a suite of multimedia resources that support the patient to develop an individualised RTW and rehabilitation plan tailored to their needs. It involves them in decisions about their care and RTW and provides a framework for their healthcare team to provide support and advice. The intervention also includes an RTW coordinator (RTWC), to facilitate active delivery of each of the elements of the intervention. This aligns with previous studies that demonstrate that the provision of an RTWC is positively associated with time to RTW and the probability of returning to work across a variety of healthcare settings.^18^,^23^ Moreover, a systematic review recommended service coordination as a core component of RTW interventions.^24^

## Aim and objectives

The aim of this trial is to assess whether an occupational support intervention for people undergoing elective primary hip or knee replacement, initiated prior to surgery, is effective in supporting a reduced time to full, sustained RTW compared with usual care and is a cost-effective use of NHS resources.

The objectives are:

1. Undertake a multicentre, two-arm parallel group randomised controlled superiority trial to determine whether a tailored occupational support intervention initiated prior to elective hip and knee replacement reduces time to ‘full’ sustained RTW.
2. Undertake a 6-month internal pilot to confirm the feasibility of the trial in terms of site set-up, recruitment rate and fidelity of intervention delivery.
3. Undertake an analysis of secondary outcomes.
4. Undertake a cost-utility and cost-effectiveness analysis of the occupational support intervention compared with usual care.
5. Assess the fidelity of intervention delivery and its acceptability to patients, health professionals and commissioners.
6. Develop an implementation plan for delivery of the intervention post-trial (depending on findings).

## Methods and analysis

The OPAL trial incorporates a clinical trial, process evaluation and economic evaluation.

### Trial design

**Figure 1:**
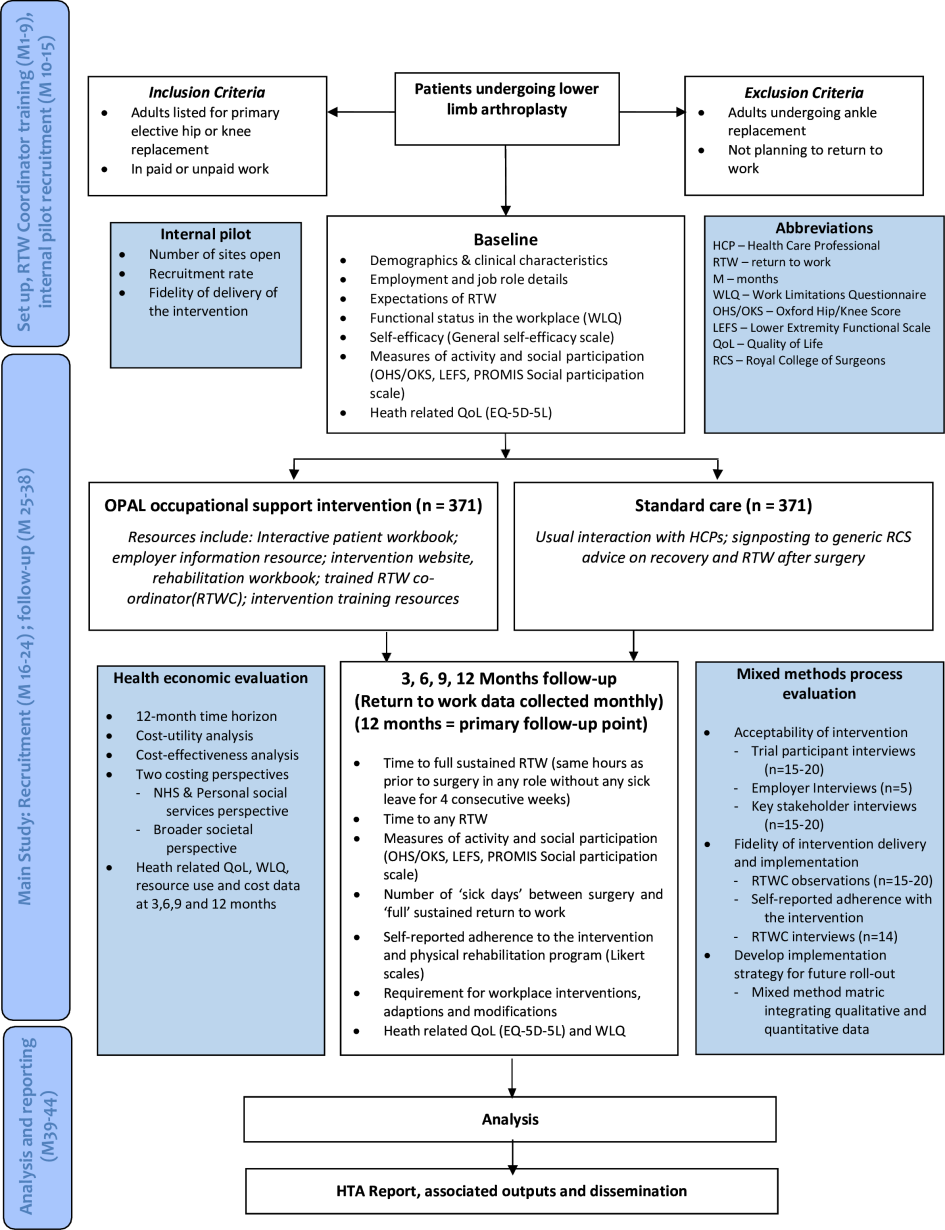
Overview of trial design and flow of participants through the trial. EQ-5D-5L, Euroqol 5 Dimensions 5 Levels; HTA, health technology assessment; NHS, National Health Service; PROMIS, Patient-Reported Outcomes Measurement Information System; QoL, Quality of Life; RTW, return to work; WLQ, Work Limitations Questionnaire

The OPAL trial is a two-arm multicentre, randomised, superiority trial with parallel groups. There will be a 6-month internal pilot and embedded economic and process evaluations. The trial will assess the effect on time to full, sustained RTW of the OPAL occupational support intervention versus usual care in 742 people undergoing elective primary hip and knee replacement in the UK. Participants will be followed-up at 3, 6, 9 and 12 months post-surgery. The participant flowchart can be seen in figure 1. Recruitment is planned to start in April 2023 and finish in July 2024.

### Trial setting

A minimum of 14 UK NHS hospitals will participate as study sites. The sites are geographically spread and include several situated within the top decile for UK deprivation. Site selection will be targeted to ensure sampling is from a diverse range of demographic and occupational groups to optimise equality, diversity and inclusion. The study interventions will commence and be delivered in secondary care. There will be interaction with primary care (GP) and commercial (employers) stakeholders as part of the intervention.

### Eligibility criteria

#### Inclusion criteria

1. Adults aged 16 years or older listed for elective primary hip or knee replacement.
2. In paid or unpaid work.
3. Intend to RTW after surgery.
4. Any hip or knee procedure that is covered by the NICE NG157 guideline and would mandate the completion of a National Joint Registry (NJR) primary hip or knee replacement data form (NJR K1 or H1 form).

#### Exclusion criteria

1. Patients undergoing emergency arthroplasty (eg, for trauma).
2. Adults listed for elective ankle replacement.
3. Adults planned to undergo further surgery within 6 months after their joint replacement.
4. Patients listed for bilateral knee replacements.

### Recruitment and informed consent

Potential participants will be identified from the hip and knee replacement waiting lists at each site. Eligibility screening for work status will take place either during the patient’s preoperative assessment clinical appointment or via phone prior to surgery. If a patient is deemed to be ineligible for the trial, the treating clinician will thank the patient for their interest but inform them verbally that they are not able to take part. They will then continue with their usual care. The patient may be informed about the trial in advance of their preoperative assessment clinical appointment where local practices allow for the provision of pre-appointment information. Potential participants will be provided with information about the trial, including a patient information sheet. Patients will have the opportunity to ask questions of the clinical and local research team before consent for the trial is obtained. If the individual wishes to take part in the trial, informed consent will be taken.

Written consent for participation may occur during the clinical appointment, remotely following the clinic (if the patient prefers further time for consideration) or remotely where the patient is identified and approached directly from the surgical waiting list. Participants will be able to provide consent electronically via Research Electronic Data Capture (REDCap), a secure, cloud-based data collection service or they may request a paper form to give consent (online supplemental appendix 1).

### Baseline assessment

After consent has been given, baseline data will be collected according to the schedule in table 1.

**Table 1:** Measurements and time points for trial outcomes

	Baseline(preop)	Monthly(postop)	After patient has RTW(postop)	3 months(postop)	6 months(postop)	9 months(postop)	12 months(postop)
Primary outcome (time to full, sustained RTW)		X					
Any RTW		X					
Workplace adaptations and modifications			X				
Demographics	X						
Oxford hip/knee score	X			X	X	X	X
Lower extremity functional scale	X			X	X	X	X
PROMIS social participation	X			X	X	X	X
EQ-5D-5L	X			X	X	X	X
Work Productivity Questionnaire	X			X	X	X	X
Health resource use	X			X	X	X	X
Patient-reported adverse events		X	X	X	X	X	X
Participant adherence and satisfaction (intervention group only)			X				

EQ-5D-5LEuroqol 5 Dimensions 5 LevelsPROMISPatient-Reported Outcomes Measurement Information SystemRTWreturn to work

### Randomisation

Following consent and baseline procedures an authorised, delegated team member at the recruiting site will access REDCap, to obtain the participant’s randomised treatment allocation. An independent statistician at York Trials Unit (YTU), who is not involved in the recruitment of participants, will generate the allocation sequence (to ensure allocation concealment). Allocation will be on a 1:1 ratio (intervention:control) and stratified by surgical site (hip or knee joint) with randomly permuted blocks of randomly varying size.

### Trial treatments

All patients recruited to the OPAL trial receive their usual clinical care as per their local sites standard hip and knee replacement pathway. All patients receive their hospital’s standard preoperative and postoperative assessments and postoperative rehabilitation. In addition to usual care, half of the participants will be randomised to receive the OPAL programme (trial intervention) as described below.

### The OPAL intervention

The aim of the OPAL intervention is to support a safe and sustained RTW after hip and knee replacement. The OPAL intervention contains two complementary elements that provide all the core components of a ‘successful’ occupational intervention for the target population, as demonstrated in the feasibility study.^3^ The two elements are:

1. Provision of multimedia information resources that support key aspects of RTW.
  - Patient workbook, which aids patients to develop their RTW plan.
  - Employer workbook, which informs employers how to support employees in their RTW.
  - Rehabilitation workbook, which helps patients tailor their exercise programme based on their work and offers a way for patients to track their exercises.
  - OPAL website multimedia resources and rehabilitation videos.
2. RTWC who provides 1:1 support and encourages engagement with and understanding of the provided information resources.

The intervention is designed to accommodate the heterogeneity of paid and unpaid occupations. All the intervention components are required to enable the delivery of a sustainable occupational intervention for this diverse population across a variety of NHS settings that deliver orthopaedic services. Participants only complete the elements of the programme that are relevant/appropriate for them.

The RTWC role facilitates the delivery of the OPAL programme. The RTWCs support the provision of education and support, provide vocational counselling and guidance, signpost to relevant resources and support multidisciplinary team involvement in the RTW process. The 1:1 nature of the interaction between the patient and the coordinator allows for individualised support and helps to ensure RTW is managed sensitively and sympathetically without placing undue pressure on patients to return. The RTWC role aligns with previous research that demonstrates the provision of a coordinator role is positively associated with time to RTW and the probability of return across a variety of healthcare settings.^19 20 22 23^

The role will be adopted by a member of the hospital orthopaedic team, such as nurses, occupational therapists and physiotherapists who will be trained in delivering the intervention. A local hospital orthopaedic team member is best placed to adopt this role due to their knowledge of local treatment pathways, rehabilitation services and the specific needs of their local patient population.

As part of the intervention, the RTWC will contact all patients prior to surgery to review and support their engagement with the information resources and provide vocational advice to aid the development of the RTW plan. They will encourage patients to share the employer booklet and their plan for returning to work with their employer. They remain a point of contact for advice and support post-surgery up to the point the patient returns to work.

Current care is extremely varied in its timing, content, format and mode of delivery^2^ and this presents challenges for the implementation and adoption of a new intervention. The OPAL intervention has been specifically designed to enable implementation across the NHS by allowing flexible delivery within the overarching framework of the intervention’s patient and staff performance objectives to maintain integrity. Delivery of the intervention will be initiated prior to surgery and continue until the patient has either returned to work or until 12 months after surgery (end-of-trial follow-up).

### Control arm

Participants in the control group will be signposted to generic RTW advice and support available via the Royal College of Surgeons of England.^25^

### Data collection

A summary of all data collection is presented in table 1. Data will be collected at baseline (pre-surgery) and 3, 6, 9 and 12 months after randomisation for the patient-report outcome measures (PROMs). Baseline data will include a collection of the individual’s work pattern pre-surgery. The RTW data will be collected monthly until participants have had a fully sustained RTW as defined in the primary outcome. Participants will receive a £10 gift voucher on completing the final trial questionnaire.

### Outcomes

#### Primary outcome

The primary outcome of the OPAL trial is time until ‘full’ sustained return to any work, defined as work resumption to the same hours as prior to joint replacement, in any role, without any day of sick leave for a consecutive 4-week period.

#### Secondary outcomes

- Time to any RTW.

- Measures of functional recovery to daily activities and social participation:

- Oxford Hip/Knee Score (OKS/OHS)^26 27^—joint-specific, PROMs designed to assess disability in patients undergoing hip (OHS) or knee (OKS) replacement. Each score contains 12 questions scored on a 5-point scale (0–4 points) producing scores ranging from 0 (poor function) to 48 (good function).
- Lower Extremity Functional Scale (LEFS)^28^—a valid self-reported patient-rated outcome for the measurement of general lower extremity function. It contains 20 questions each scored on a 5-point scale (0–4 points) producing scores ranging from 0 (very low function) to 80 (very high function). It has been shown to have good measurement properties compared with the SF36 and WOMAC scores^29^—WOMAC (Western Ontario and McMaster University Osteoarthritis Index) is disease specific for hip or knees and is scored by the patient. SF36 (Short Form 36 Health Survey Questionnaire) is used to indicate the health status of participant populations.
- PROMIS social participation short form questionnaires (eg, ability to participate questionnaire, satisfaction with social roles and activities questionnaire, satisfaction with participation in social roles questionnaire)^30 31^—PROMIS (Patient-Reported Outcomes Measurement Information System) is a set of person-centred measures that evaluates and monitors physical, mental and social health. We will use the social health tools developed by PROMIS to measure social participation and satisfaction with participation and social roles.

- Number of ‘sick days’ between surgery and ‘full’ sustained RTW.

- Participant adherence to the intervention and the intervention’s physical rehabilitation programme (including self-reported, 5-point Likert scales (very helpful, helpful, neither helpful nor unhelpful, unhelpful, very unhelpful)).

- Proportion of participants using workplace interventions, adaptations and modifications to facilitate their RTW (eg, changes in working hours and shift patterns, changes to work role or work environment or use of additional equipment within the workplace).

- Health-related quality of life (Euroqol 5 Dimensions 5 Levels (EQ-5D-5L)).^32^

- Work productivity (Work Limitations Questionnaire).^33 34^

### Data storage

REDCap will be used to capture all trial data electronically. To minimise attrition, we will use multiple methods to keep in touch with participants.

Data will be held securely on the cloud-hosted REDCap server. Access to the trial interface will be restricted to named authorised individuals granted user rights by a REDCap administrator at YTU. All trial files will be stored in accordance with Good Clinical Practice (GCP) guidelines. Trial documents (paper and electronic) held at YTU will be retained in a secure location for the duration of the trial. All work will be conducted using the University of York’s data protection policy which is publicly available.

### Follow-up and withdrawal

Participants will be followed-up at 3, 6, 9 and 12 months, as well as monthly for a maximum of 12 months or until the primary outcome is achieved. Participants will be sent a maximum of two reminders via their preferred method of contact to complete their questionnaires, with a final attempt to obtain data by telephone.

Participants can withdraw from the trial at any point during the trial by directly contacting the trial team at YTU, or their clinical team. If a participant indicates that they wish to withdraw from the trial, they will be asked whether they wish to withdraw from the intervention only (ie, withdrawal from engaging with the RTWC and workbooks) or withdraw fully from the trial. Where withdrawal is only from the intervention then follow-up data will continue to be collected. Participants will be informed that they do not have to give a reason for their decision to withdraw from the trial. However, if the participant indicates the reason this will be recorded. Data provided by participants who withdraw will be retained for analysis up until the point of withdrawal—as detailed in the patient consent.

### Confidentiality

The research teams will hold data according to the General Data Protection Regulation (May 2018). Each participant will be allocated a unique trial identification number, and on all trial-specific documents, other than the signed consent form, the participant will be referred to by the participant number, not by name. Only relevant members of the trial team will have access to participant personal information that will be stored on REDCap.

### Sample size

A previous meta-analysis of Randomised Controlled Trials (RCTs) examining work coordination programmes for work disability found an HR for time to RTW of 1.34 (95% CI: 1.14 to 1.56).^23^ With 90% power, 5% alpha, to detect an HR=1.34, assuming a median time to RTW of 3.2 months.^17^ Accounting for 20% attrition, an average of 31 patients per RTWC, intraclass correlation (ICC)=0.01 the sample size required is 742 with equal allocation. A minimum of 12 RTWC would be required to deliver the trial. The sample size was calculated for a log-rank test using PS power and Sample Size software.

### Data and statistical analysis

Statistical analyses will be on intention-to-treat (ITT) basis with patients being analysed in the groups to which they were randomised. Statistical significance will be at the 5% level and analyses will be conducted in the latest available version of Stata or similar statistical software.

Baseline characteristics will be reported descriptively by treatment group. Continuous data will be summarised as means, SD, medians and ranges and categorical data will be summarised as frequencies and percentages. No formal statistical comparisons of baseline data will be undertaken. Data will be visually inspected, and any imbalance reported.

### Primary outcome

The primary analysis will be an assessment of treatment differences evaluated using the Cox proportional hazard model with shared centre and RTWC frailty effects and adjusting for important baseline covariates (including stratification factors). The HR, CI and p value will be reported. Median time until full return to any work and Kaplan-Meier survival curves will be presented by the trial arms and a log-rank survival comparison will be made.

### Secondary outcomes

Time to any RTW will be analysed using a similar model to the primary analysis. Other secondary outcomes will be analysed using linear mixed models (eg, OKS/OHS), LEFS, PROMIS) or logistic regression (workplace intervention) as appropriate. For the number of sick days taken after surgery and before sustained RTW, a Poisson regression will be completed. Differences between allocated groups will be reported for all available time points.

### Process evaluation

A mixed-methods process evaluation will be used to assess the intervention using a revised version of the Carroll *et al*^35^ conceptual framework for implementation fidelity. To inform how the trial findings could be incorporated into developments in service delivery/future implementation, we will draw on relevant data from across the qualitative components of the trial, including how the intervention was implemented across the trial sites. This will be summarised using normalisation process theory (NPT)^36^ and, together with the main trial findings on effectiveness and cost-effectiveness data, will be discussed at the second stage interviews with service leaders. Data will be used to develop an implementation strategy for future roll out across the NHS, if appropriate.

### Intervention fidelity and qualitative outcomes

The following data will be collected:

- Qualitative observations of the RTWC during an initial appointment.

- An intervention delivery checklist was completed for all trial participants.

- Participant outcome questionnaires (3, 6, 9 and 12 months) and self-reported adherence to the intervention.

- Completion rates of patient/rehabilitation workbooks.

- Qualitative interviews with trial participants from the intervention arm.

- Qualitative interviews with trial participants’ employers.

- Qualitative interviews with RTWCs at the start and end of the intervention period.

- Qualitative interviews with the service leaders at the start and end of the intervention period.

Qualitative sampling will be purposive to achieve maximum variation^37^ and adequate information power.^38^

We will use NVivo software to assist qualitative data organisation and coding. We will conduct a framework analysis (using broad categories as described in the implementation fidelity model and the key characteristics of NPT) to summarise findings according to key trial outcomes: intervention fidelity, acceptability of intervention, engagement with/adherence to the intervention and implementation.^39^ Descriptive statistics of the quantitative process evaluation data will be integrated with qualitative findings using a mixed method matrix. Where relevant these data will be integrated with appropriate quantitative data to provide a more complete picture.

### Economic evaluation

Enhancing evidence on the cost-effectiveness of occupational therapies is highlighted in both the 2007 and 2020 research priorities of the Royal College of Occupational Therapists.^40^ However, existing systematic reviews indicate a lack of studies in the literature that report on the cost-effectiveness of occupational therapies.^41^ A full economic evaluation will be conducted alongside the OPAL trial to assess the cost-effectiveness of the OPAL intervention versus usual care over a 12-month period. The base case analysis will be performed from an NHS and personal social services perspective and follow the principle of ITT. A secondary analysis will explore the wider societal perspective.

Cost estimates for the intervention will incorporate the cost of all associated resources and materials required for its development, training and delivery. Resource use questions in the follow-up case report forms will capture participants’ healthcare utilisation in relation to their replaced hip or knee across primary, secondary and community care settings. Unit costs, obtained from established national costing sources, will be applied to each resource use item to estimate the total cost per participant.^33 42^ For the secondary analysis, data on lost productivity will be collected using the Work Limitations Questionnaire.^33^

The primary health outcome measure in the economic evaluation is quality-adjusted life-year (QALY), which will be calculated based on data collected from the EQ-5D-5L questionnaire at baseline and at each follow-up.^32 43^ An incremental cost-effectiveness analysis will be conducted to compare the intervention with usual care. Incremental cost-effectiveness ratios (ICERs) will be calculated and assessed against NICE’s willingness to pay (WTP) thresholds of £20 000 to £30 000 per QALY.^44^ In addition, the incremental cost per missed workday averted will also be calculated.

Uncertainty around the calculated ICERs will be assessed using a non-parametric bootstrapping technique.^45^ The bootstrapping results will be used to generate cost-effectiveness acceptability curves to illustrate the probability of the OPAL intervention being cost-effective at different WTP thresholds.^46^ Rubin’s multiple imputation method will be adopted to handle missing data, assuming the data is missing at random.^47 48^ Sensitivity analyses will be conducted to explore the robustness of the cost-effectiveness findings. A health economic analysis plan will be developed prior to data analysis and will follow the latest NICE health technology evaluations manual.^44^

### Plans to give access to the full protocol, participant level-data and statistical code

This paper constitutes a complete representation of the trial protocol. The full protocol and related documents for the OPAL trial are available (insert where documents are kept, eg, National Institute for Health and Care Research (NIHR) website). Anonymised participant-level data may be requested from YTU after trial completion. The approval of any data requests is at the discretion of the chief investigator and YTU.

### Oversight and monitoring

The coordination of the OPAL trial will be managed by YTU in collaboration with the sponsor and chief investigator. The Trial Management Group (TMG) will monitor the day-to-day management of the trial.

A Trial Steering Committee (TSC) will monitor the progress of the trial, provide independent advice and the independent chair will make recommendations to the funder.

An independent Data Monitoring Committee (DMC) will monitor the data arising from the trial and make recommendations to the TSC about trial continuation based on ethical and safety considerations. The trial will also be monitored by the sponsor (South Tees NHS Foundation Trust) and a representative will be invited to attend the TMG, TSC and DMC.

### Adverse event reporting and harms

#### Adverse events

Adverse events are defined as any untoward medical occurrence (ie, any unfavourable and unintended sign, symptom or disease), experienced by a trial participant and which is temporarily associated with trial treatment (intervention or control) and is related to hip or knee replacement or to the trial intervention or control treatments.

For the OPAL trial, Adverse Events (AEs) will only be considered as related to the hip or knee replacement or to the trial intervention or control treatments if:

- They occur during the inpatient stay (after randomisation) for the primary joint replacement.
- They occur in the same limb as the replaced joint.
- They are related to the anaesthetic, surgery, hospital admission, physiotherapy or radiographic assessment.
- They are thought to be related to the trial intervention, trial processes.

AEs will be collected from the point of randomisation onwards, up to the 12-month follow-up point. Events occurring before randomisation will not be recorded. Ongoing review of AEs will take place during monthly TMG meetings, discussed with the TSC and reported to the sponsor and research ethics committee in-line with their guidelines.

### Patient and public involvement

The trial has been developed with patient advisors who have had hip or knee arthritis and joint replacement surgery. A patient advisory group, along with public members who worked with us securing funding for the trial, will continue to be involved during the conduct of this trial, support the development of patient facing documents, advise on trial processes and suggest how best to report trial findings to the public and patients. There are two members of the patient and public involvement group that are co-applicants of the trial.

### Ethics and dissemination

Dissemination will focus on supporting the wider adoption and implementation of the intervention (if effective); the dissemination plan, developed at the outset of the trial will be amended as the results of the implementation substudy become available. An HTA monograph of the findings will be produced as well as publications in other peer-reviewed journals, regardless of the trial outcome.

We will produce lay summaries targeted at specific stakeholders, presentations at relevant professional society events and press releases through the collaborating NHS organisations, occupational health service and universities. A plain language summary will be disseminated to trial participants who have expressed an interest in hearing about the findings.

At the end of the trial, the intervention content will be made available through the NIHR HTA journals webpage, with an implementation plan/toolkit. Even if the intervention is not proven to be effective there may be individual elements that would be useful to healthcare professionals or patients. Data will be made available to allow for inclusion in future meta-analyses with studies of the same intervention in other trials.

This trial was approved by the West Midlands—Edgbaston Research Ethics Committee, Rec Reference 23/WM/0013.

## Discussion

The OPAL trial is testing an evidence-based intervention developed as part of earlier NIHR-funded research (the OPAL feasibility study HTA 15/28/02). This intervention supports the implementation of recently developed NICE guidance (NG157) and will contribute to reducing variation in the provision of occupational advice and the support patients receive to enable their RTW after hip and knee replacement surgery. Encouraging and supporting RTW through an occupational intervention initiated prior to surgery could help minimise the health and socioeconomic consequences of joint replacement surgery. There is also potential for this intervention to be adapted and implemented in other surgical settings, using the framework for delivery and adoption developed during this trial. Such wider applicability will increase the impact of the trial from both a patient and societal perspective.

If successful, the intervention has the potential to greatly improve how patients undergoing hip and knee replacement are supported in their return to paid and unpaid work. It will also provide key data on the clinical and cost-effectiveness of delivering occupational support in this setting.

## supplementary material

10.1136/bmjopen-2024-085962 online supplemental file 1

## References

[1] NICE (2020). Joint replacement (primary): hip, knee and shoulder. https://www.nice.org.uk/guidance/ng157.

[2] Tsang B, McDonald D, McNamara I (2020). National survey of occupational advice for lower limb arthroplasty patients. Occup Med (Lond).

[3] Baker P, Coole C, Drummond A (2020). Occupational advice to help people return to work following lower limb arthroplasty: the OPAL intervention mapping study. Health Technol Assess.

[4] Coole C, Nouri F, Narayanasamy M (2021). Total hip and knee replacement and return to work: clinicians’ perspectives. Disabil Rehabil.

[5] Nouri F, Coole C, Baker P (2020). Return to work advice after total hip and knee replacement. Occup Med (Lond).

[6] Nouri F, Coole C, Narayanasamy M (2019). Managing Employees Undergoing Total Hip and Knee Replacement: Experiences of Workplace Representatives. J Occup Rehabil.

[7] The Scottish Arthroplasty Project (2021). Scottish arthroplasty project report. https://www.arthro.scot.nhs.uk/Reports/Main.html.

[8] National Joint Registry NJR 17th annual report 2020. https://www.arthro.scot.nhs.uk/Reports/Main.html.

[9] Morgan OJ, Hillstrom HJ, Ellis SJ (2019). Osteoarthritis in England: Incidence Trends From National Health Service Hospital Episode Statistics. *ACR Open Rheumatol*.

[10] Kurtz SM, Lau E, Ong K (2009). Future young patient demand for primary and revision joint replacement: national projections from 2010 to 2030. Clin Orthop Relat Res.

[11] Waddell G, Burton K (2006). IS WORK GOOD FOR YOUR HEALTH AND WELL-BEING?.

[12] Gignac MAM, Badley EM, Lacaille D (2004). Managing arthritis and employment: making arthritis-related work changes as a means of adaptation. Arthritis Rheum.

[13] O’Brien GE, Feather NT (1990). The relative effects of unemployment and quality of employment on the affect, work values and personal control of adolescents. J Occup Psychol.

[14] Sankar A, Davis AM, Palaganas MP (2013). Return to work and workplace activity limitations following total hip or knee replacement. Osteoarthr Cartil.

[15] Kleim BD, Malviya A, Rushton S (2015). Understanding the patient-reported factors determining time taken to return to work after hip and knee arthroplasty. Knee Surg Sports Traumatol Arthrosc.

[16] Tilbury C, Leichtenberg CS, Tordoir RL (2015). Return to work after total hip and knee arthroplasty: results from a clinical study. Rheumatol Int.

[17] Tilbury C, Schaasberg W, Plevier JWM (2014). Return to work after total hip and knee arthroplasty: a systematic review. Rheumatology (Oxford).

[18] Severens JL, Mulder J, Laheij RJ (2000). Precision and accuracy in measuring absence from work as a basis for calculating productivity costs in The Netherlands. Soc Sci Med.

[19] Skarpaas LS, Haveraaen LA, Småstuen MC (2019). The association between having a coordinator and return to work: the rapid-return-to-work cohort study. BMJ Open.

[20] Shaw W, Hong QN, Pransky G (2008). A literature review describing the role of return-to-work coordinators in trial programs and interventions designed to prevent workplace disability. J Occup Rehabil.

[21] Varatharajan S, Côté P, Shearer HM (2014). Are work disability prevention interventions effective for the management of neck pain or upper extremity disorders? A systematic review by the Ontario Protocol for Traffic Injury Management (OPTIMa) collaboration. J Occup Rehabil.

[22] Franche R-L, Cullen K, Clarke J (2005). Workplace-based return-to-work interventions: a systematic review of the quantitative literature. J Occup Rehabil.

[23] Schandelmaier S, Ebrahim S, Burkhardt SCA (2012). Return to work coordination programmes for work disability: a meta-analysis of randomised controlled trials. PLoS One.

[24] Hoefsmit N, Houkes I, Nijhuis FJN (2012). Intervention characteristics that facilitate return to work after sickness absence: a systematic literature review. J Occup Rehabil.

[25] Royal College of Surgeons of England (2022). Helping you to make a speedy recovery after total hip replacement. https://www.rcseng.ac.uk/patient-care/recovering-from-surgery/total-hip-replacement/returning-to-work/.

[26] Dawson J, Fitzpatrick R, Carr A (1996). Questionnaire on the perceptions of patients about total hip replacement. J Bone Joint Surg Br.

[27] Dawson J, Fitzpatrick R, Murray D (1998). Questionnaire on the perceptions of patients about total knee replacement. J Bone Joint Surg Br.

[28] Binkley JM, Stratford PW, Lott SA (1999). The Lower Extremity Functional Scale (LEFS): scale development, measurement properties, and clinical application. Phys Ther.

[29] Pua YH, Cowan SM, Wrigley TV (2009). The Lower Extremity Functional Scale could be an alternative to the Western Ontario and McMaster Universities Osteoarthritis Index physical function scale. J Clin Epidemiol.

[30] Hahn EA, Beaumont JL, Pilkonis PA (2016). The PROMIS satisfaction with social participation measures demonstrated responsiveness in diverse clinical populations. J Clin Epidemiol.

[31] HealthMeasures (2022). PROMIS® (patient-reported outcomes measurement information system). https://www.healthmeasures.net/explore-measurement-systems/promis.

[32] EuroQol Research Foundation (2022). EQ-5d user guides 2022. https://euroqol.org/publications/user-guides/.

[33] Walker TJ, Tullar JM, Diamond PM (2017). Validity and Reliability of the 8-Item Work Limitations Questionnaire. J Occup Rehabil.

[34] Lerner D, Amick BC, Rogers WH (2001). The Work Limitations Questionnaire. Med Care.

[35] Hasson H (2010). Systematic evaluation of implementation fidelity of complex interventions in health and social care. Implement Sci.

[36] May C, Finch T (2009). Implementing, Embedding, and Integrating Practices: An Outline of Normalization Process Theory. Sociology.

[37] Patton MQ (2002). Qualitative Research and Evaluation Methods.

[38] Malterud K, Siersma VD, Guassora AD (2016). Sample Size in Qualitative Interview Studies: Guided by Information Power. Qual Health Res.

[39] Gale NK, Heath G, Cameron E (2013). Using the framework method for the analysis of qualitative data in multi-disciplinary health research. BMC Med Res Methodol.

[40] (2021). Identifying research priorities for occupational therapy in the UK.

[41] Weatherly H, Davies C (2021). Economic evaluation of OT services: Guidance and opportunities. Br J Occup Ther.

[42] Jones K, Weatherly H, Birch S (2022). Unit costs of health and social care 2022 manual. https://www.pssru.ac.uk/project-pages/unit-costs/.

[43] Richardson G, Manca A (2004). Calculation of quality adjusted life years in the published literature: a review of methodology and transparency. Health Econ.

[44] NICE (2022). NICE Health Technology Evaluations: The Manual.

[45] Efron B, Tibshirani R (1993). An Introduction to the Bootstrap.

[46] Faria R, Gomes M, Epstein D (2014). A guide to handling missing data in cost-effectiveness analysis conducted within randomised controlled trials. Pharmacoeconomics.

[47] Rubin DB (1987). Multiple Imputation for Nonresponse in Surveys.

[48] White IR, Royston P, Wood AM (2011). Multiple imputation using chained equations: Issues and guidance for practice. Stat Med.

