# Supplementary material for: Occupational support following arthroplasty of the lower limb (OPAL): trial protocol for a UK-wide phase III randomised controlled trial
Source: BMJ Open. 2024 Sep 16;14(9):e085962. doi: 10.1136/bmjopen-2024-085962 (PMC11409380; doi:10.1136/bmjopen-2024-085962)
Supplement: online supplemental file 1 [file bmjopen-14-9-s001.pdf]

UNIVERSITY of York

OPAL
Occupational support for Patients undergoing Arthroplasty of the Lower limb

Insert trust logo

PATIENT CONSENT FORM
OPAL

Participant ID number: [Insert participant ID number]

Please initial each box

1. I confirm that I have read and understand the information sheet version [XX] dated [XX/XX/XXXX] for the above study and have had the opportunity to ask questions about the study and any questions have been answered to my satisfaction. — Initials

2. I understand that my participation is voluntary and that I am free to withdraw consent for participation at any time without giving any reason, without my medical care or legal rights being affected. I understand that if I withdraw from the study, any data provided up to that point will still be used. — Initials

3. I understand that relevant sections of my medical notes and data collected during the study may be looked at by individuals from the University of York, and other members of the research team, regulatory authorities, or from the NHS Trust, where it is relevant to taking part in this research. This will be done in accordance with the General Data Protection Regulation (GDPR) and the necessary data sharing agreements. I give permission for these individuals to access my records. — Initials

4. I agree to York Trials Unit at the University of York holding my personal details and consent form, to allow them to send me questionnaires and other OPAL study related documents, and to allow them to contact me about queries relating to the study. — Initials

5. I agree to my General Practitioner being informed of my participation in the study and being advised of any significant information relating to my health that comes to light during my participation. — Initials

6. I understand that the information collected about me will be used to support other research in the future and may be shared anonymously with other researchers. — Initials

7. **I agree to take part in the OPAL study and that data will be collected from me in relation to this study through questionnaires.** — Initials

Funded by NIHR

PLEASE TURN OVER

In addition to the above statements, please initial any of the following additional **optional** statements you agree with. Please leave blank if you disagree. Your participation in the OPAL study will not be affected if you do not agree to any of these.

Please initial each box

8. I am willing to receive an invitation to take part in a one-to-one interview to share my experiences about taking part in this study and for my contact details to be shared with members of the research team based at [XXXXXX]. — Initials

9. I am willing to receive emails in connection with the OPAL study that provide links to electronic versions of the study questionnaires. — Initials

10. I understand that my email address will be shared with a third party (REDCap [Research Electronic Data Capture]) who will send study emails. My email address and replies to emails will be held by them and the University of York in accordance with the General Data Protection Regulation (GDPR) and the Data Protection Act (DPA) 2018 and will not be shared with other parties outside of the OPAL study. — Initials

11. I give consent to link data collected from the study to routinely collected health data stored in national databases (via your NHS number), and to share this information anonymously with other researchers. I understand my personal details will not be provided to anyone else or used for any other purpose. — Initials

Please sign below to confirm you continue to agree to participate in the OPAL study after discussing it with the research team:

	d d / m m / y y y y<br>_ _ / _ _ / 2 0 _ _	
Name of participant [please print]	Today's Date	Signature of participant
	_ _ / _ _ / 2 0 _ _	
Name of person taking consent [please print]	Today's Date	Signature of person taking consent

[Filing: one copy to participant, one copy in site file, one copy to York Trials Unit]

Funded by NIHR
