# Supplementary material for: Occupational support following arthroplasty of the lower limb (OPAL): trial protocol for a UK-wide phase III randomised controlled trial
Source: BMJ Open. 2024 Sep 16;14(9):e085962. doi: 10.1136/bmjopen-2024-085962 (PMC11409380; doi:10.1136/bmjopen-2024-085962)
Supplement: Supplementary file 2 [file bmjopen-2024-085962.reviewer_comments.pdf]

PEER REVIEW HISTORY

BMJ Open publishes all reviews undertaken for accepted manuscripts. Reviewers are asked to complete a checklist review form and are provided with free text boxes to elaborate on their assessment. These free text comments are reproduced below.

ARTICLE DETAILS

**TITLE (PROVISIONAL)**	Occupational support following Arthroplasty of the lower limb (OPAL): trial protocol for a UK wide phase III randomised controlled trial
**AUTHORS**	Sheehan, Lucy; Woodward, Amie; Archer, Marion; Jordan, Carol; Martland, Maisie; McDonald, David; Parkinson, Gill; Watkins, Lou; Adamson, Joy; Drummond, Avril; Hewison, Ann; Keding, Ada; Kottam, Lucksy; MADAN, IRA; McDaid, Catriona; Reed, Mike; Sinclair, Lesley; Smith, Toby; Thomson, Louise; Wu, Qi; Baker, Paul

VERSION 1 - REVIEW

**REVIEWER NAME**	Farivar, Abdolhay
**REVIEWER AFFILIATION**	Royal North Shore Hospital, Rheumatology Department
**REVIEWER CONFLICT OF INTEREST**	No competing interest
**DATE REVIEW RETURNED**	04-May-2024

**GENERAL COMMENTS**	It is a well-designed protocol paper. However, as you know, you may see lots of challenges during the implementation of the intervention. Hope all goes well for you!

**REVIEWER NAME**	Arnold, Susanne
**REVIEWER AFFILIATION**	Warwick Medical School, Warwick Clinical Trials Unit
**REVIEWER CONFLICT OF INTEREST**	No competing interests
**DATE REVIEW RETURNED**	13-May-2024

**GENERAL COMMENTS**	This is a review of a trial protocol for Occupational support following Arthroplasty of the lower limb (OPAL).<br><br>Abstract: clear overview of the background to the problem and the plan for the trial.<br><br>Introduction: important rationale and plan for the trial described clearly and in detail<br><br>Methods:<br>Page 8, lines 224 - 227: Could you just give a few examples of what is included in each of these workbooks i.e. what is that the participants have to actually complete/do.<br><br>Page 9 and table 1: Why are you only collecting adverse events at 6 and 12 months?<br><br>Page 10, line 310: You mention adherence to the intervention but do

	not give any detail that I can see about how you will actually measure this or what you mean by adherence. And along with this can I ask, if the patient does not get back to work without any sick days in four weeks in the 12-month follow-up period how will this be interpreted?<br><br>Page12, qualitative section: You have included an intervention delivery checklist, participant questionnaires and completion rates in this qualitative section - these are not qualitative measures.<br><br>Discussion: a clear overview

VERSION 1 – AUTHOR RESPONSE

Methods:

Page 8, lines 224 - 227: Could you just give a few examples of what is included in each of these workbooks i.e. what is that the participants have to actually complete/do. Thank you for the comment. We have now amended this on page 8, lines 225-230.

Page 9 and table 1: Why are you only collecting adverse events at 6 and 12 months? Thank you for the comment. We have amended this on page 7, table 1.

Page 10, line 310: You mention adherence to the intervention but do not give any detail that I can see about how you will actually measure this or what you mean by adherence. And along with this can I ask, if the patient does not get back to work without any sick days in four weeks in the 12-month follow-up period how will this be interpreted? Thank you for the comment. The authors have noted in lines 315-316 that adherence will be self-reported, including adherence to the rehab programme. This is collected in the primary outcome achieved questionnaire. Likert scales will also be employed to measure how helpful the participants found the intervention. If a patient does not return to work (as per the criteria) in a 12-month period, this will form part of the analysis of the effectiveness of the intervention.

Page12, qualitative section: You have included an intervention delivery checklist, participant questionnaires and completion rates in this qualitative section - these are not qualitative measures. Thank you for your comment. We have amended this on page 12, line 406.
